# Granuloma Formation in Pulmonary Sarcoidosis

**DOI:** 10.3389/fimmu.2013.00437

**Published:** 2013-12-10

**Authors:** Caroline E. Broos, Menno van Nimwegen, Henk C. Hoogsteden, Rudi W. Hendriks, Mirjam Kool, Bernt van den Blink

**Affiliations:** ^1^Department of Pulmonary Medicine, Erasmus MC, Rotterdam, Netherlands

**Keywords:** pulmonary sarcoidosis, granuloma, formation, integrity, dendritic cells, T helper 1 cells, T helper 17 cells, regulatory T cells

## Abstract

Sarcoidosis is a granulomatous disorder of unknown cause, affecting multiple organs, but mainly the lungs. The exact order of immunological events remains obscure. Reviewing current literature, combined with careful clinical observations, we propose a model for granuloma formation in pulmonary sarcoidosis. A tight collaboration between macrophages, dendritic cells, and lymphocyte subsets, initiates the first steps toward granuloma formation, orchestrated by cytokines and chemokines. In a substantial part of pulmonary sarcoidosis patients, granuloma formation becomes an on-going process, leading to debilitating disease, and sometimes death. The immunological response, determining granuloma sustainment is not well understood. An impaired immunosuppressive function of regulatory T cells has been suggested to contribute to the exaggerated response. Interestingly, therapeutical agents commonly used in sarcoidosis, such as glucocorticosteroids and anti-TNF agents, interfere with granuloma integrity and restore the immune homeostasis in autoimmune disorders. Increasing insight into their mechanisms of action may contribute to the search for new therapeutical targets in pulmonary sarcoidosis.

## Introduction

Sarcoidosis is a granulomatous disorder of unknown cause, affecting multiple organs, but mainly the lungs. In 10–30% of the cases, sarcoidosis becomes chronic and progressive leading to debilitating disease and sometimes death (1). Its etiology is intriguing, since a part of its definition (i.e., *unknown cause*) makes it uniquely different from granulomatous disorders arising from exposure to a known chronically persisting antigen, such as tuberculosis, visceral leishmaniasis, and chronic beryllium disease (2, 3). Nevertheless, several observations support an antigen-induced disease etiology. First, epidemiological research identified environmental and occupational risk factors, such as exposure to musty odors and insecticides (4). Second, infectious agents, including *Propionibacterium acnes* (*P. acnes*) and *Mycobacterium tuberculosis* (*Mtb*), have been implicated, since genomes of these species are detected within sarcoid granulomas (5). A role for mycobacterial peptides is further supported by the presence of T lymphocytes that are highly responsive toward 6-kDa early secreted antigenic protein (ESAT-6) or catalase peroxidase (KatG) in the broncho-alveolar lavage fluid (BALF) of sarcoidosis patients (6– 8). Third, a limited clonality of CD4^+^ T cells, expressing the AV2S3 T cell receptor, was demonstrated within the lungs of HLA-DRB1*03 positive sarcoidosis patients, which is consistent with an antigenic response (9–12). Finally, evidence for an antigen-induced disease lies within the granulomatous reaction that is virtually indistinguishable from sarcoid granulomas and occurs in individuals with sarcoidosis upon subcutaneous injection of homogenates from allogeneic sarcoid spleen or lymph nodes (LNs), i.e., the Kveim–Siltzbach test (13, 14).

## Genetic Risk Factors in Sarcoidosis

People all over the world suffer from sarcoidosis (15). Familial clustering (16), increased concordance in monozygotic twins (17) and variations in susceptibility and disease presentation among different ethnic groups (18), suggest the importance of genetic, next to environmental risk factors in the etiology.

Genome-wide association studies (GWAS) identified polymorphisms within genes coding for proteins involved in T cell activation, differentiation, proliferation, and survival, including NOTCH 4 and ANXA11 (19, 20). Additionally, GWAS and case-control studies identified important genetic risk factors within the antigen presentation locus at 6p21.3, which contains genes encoding proteins involved in both antigen presentation and T cell regulation, including human leukocyte antigen (HLA) and *butyrophilin-like* protein (BTNL)-2, respectively (20–23).

Specific HLA class II antigens are associated with certain sarcoidosis disease phenotypes. For example, the HLA-DRB1*03 and DQB1*0201 alleles have been associated with an acute disease onset, Löfgren syndrome and resolving disease, whereas in contrast HLA-DRB1*15 and DQB1*0601 are associated with chronic sarcoidosis (24–27). It is conceivable that both resolving and persistent sarcoidosis arise due to a unique combination of a specific genetic background and exposure to one or several environmental triggers (28). This unique combination might lead to persistent stimulation of the immune system, contributing to granuloma formation and sustainment.

In this article we review the current knowledge on the role of the immune activation in pulmonary sarcoidosis and propose a hypothesis on the origin of granuloma formation. Secondly, we aim to discuss granuloma integrity, highlighting areas for research into new therapeutical targets.

## Granuloma Formation

A well-developed sarcoid granuloma consists of a tightly formed conglomerate of epithelioid- and multinucleated-giant cells (MGCs) encircled by lymphocytes, especially CD4^+^ T helper (Th) cells, but also rare CD8^+^ T cells and B cells (1). Both granuloma formation and integrity depend on the availability and supply of these different cells (29). The chronological order of immunological events and the exact role of these cells during the sarcoid granulomatous response remain obscure, due to the lack of an animal model for sarcoidosis. Nevertheless, careful clinical observations and in-depth research on functional properties of different cells involved provide essential information to unravel the cellular and molecular mechanisms of granuloma formation.

### Clinical signs

Cardinal features of pulmonary sarcoidosis are mediastinal lymphadenopathy, parenchymal, and airway granulomas, giving rise to upper lobe nodules in a perilymphatic or bronchovascular distribution and signs of a CD4^+^ T cell alveolitis. An interstitial pneumonitis, found on open lung biopsy, is classically thought to represent a very early stage of granuloma formation (30). Spontaneous remission and reactivation of sarcoidosis makes it difficult to ascertain the exact sequence of these cardinal features, however several findings strongly suggest a certain order in the majority of patients, which may add to the hypothesis on granuloma formation as described below.

Although it is well known that patients do not go through all disease stages as described by Scadding (from I to IV) sequentially, arguably pulmonary sarcoidosis starts in the draining LN. As stage I (bihilar lymphadenopathy) is most often asymptomatic, it is conceivable that it precedes pulmonary involvement, seen in stage II and III. Additionally, progression of stage I to II disease is well known, while development of stage I after stage III is uncommon. Finally, a recent trial found an increased diagnostic sensitivity of LN-derived fine needle aspirates, compared with transbronchial lung biopsies (31). These data suggest that the first granulomas are formed within the mediastinal LN, only later followed by granuloma formation within the lungs.

Consequently, LN-specific immune reactions are important in early sarcoid granuloma formation, such as antigen presentation by dendritic cells (DCs). DCs are the only cells capable to pick up antigens and migrate to the LN where they present antigens to naïve T cells. Hereby they initiate highly specific clonal T cell differentiation and proliferation (32). Alternatively, LN-resident DCs may encounter antigenic particles, which we propose are submicroscopic and may therefore have passively migrated through the afferent lymph. The activated and differentiated Th cells migrate toward the site of inflammation, orchestrated by chemokines.

Macrophages contribute to early recognition of the putative sarcoid antigen in the lungs, thereby attracting mononuclear cells, including monocytes and LN-activated lymphocytes. The ensuing influx of cells leads to an interstitial pneumonitis, characterized by a mixed mononuclear cell infiltrate in the alveolar wall and CD4^+^ T cell alveolitis (30).

At the site of antigen encounter, antigen-presenting cells (APCs) induce persistent stimulation of the immune response, mediated by HLA-related proteins, leading to continuous recruitment and local expansion of lymphocytes and eventually granuloma formation. The central localization of macrophages within the final epithelioid aggregate supports an important role in antigen presentation at the site of granuloma formation. Alternatively, DCs may play a critical role in antigen presentation within the granuloma. Their capacity for antigen sampling within the lymph fluid makes them likely candidates to contribute to the induction of the perilymphatic localized granulomas (33, 34).

**Figure 1:**
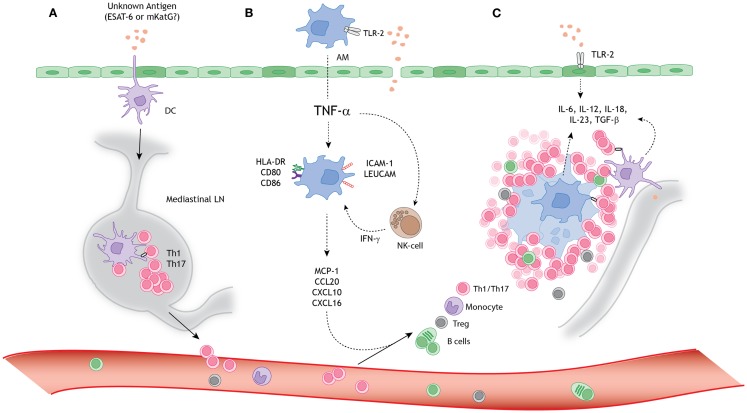
**A schematic model for granuloma formation in pulmonary sarcoidosis**. An unknown airborne-antigen activates **(A)** interstitial dendritic cells (DCs), **(B)** alveolar macrophages (AMs), and **(C)** alveolar epithelial cells type II (AEC-II) (*dark green*), simultaneously. This process is initiated by toll-like receptor-2 (TLR-2) ligands, possibly *Mycobacterium tuberculosis*-derived ESAT-6 or mKatG. **(A)** The interstitial DCs pick up the putative antigen and migrate toward the mediastinal lymph nodes (LNs), where they initiate differentiation and clonal expansion of T helper (Th)1 and 17 cells. **(B)** Simultaneously, AMs produce tumor necrosis factor-α (TNF-α), which initiates upregulation of activation (HLA-DR and CD80/86) and adhesion (ICAM-1 and LeuCAM) molecules. Macrophages produce chemokine ligands (MCP-1, CCL20, CXCL10, and CXCL16) under stimulation of both TNF-α and natural-killer (NK) cell-derived interferon-γ (INF-γ), thereby attracting Th1/17 cells, monocytes, regulatory T cells (Tregs), and B cells. **(C)** The lung environment is characterized by the presence of Th1 and Th17 favoring cytokines, such as IL-6, IL-12, IL-18, IL-23, and TGF-β, produced by macrophages, perilymphatic DCs, and AEC-II. Persistent stimulation, mediated by antigen presenting cells (APCs), leads to continuous cellular recruitment to the site of inflammation, which leads to granuloma formation. Tregs infiltrating the granuloma fail to diminish the exaggerated immune response, thereby contributing to granuloma persistence and integrity.

In the following paragraphs we describe the current knowledge on the role of macrophages, DCs, and lymphocytes in sarcoid granuloma formation in more detail, also summarized in Figure 1.

### Macrophages

Upon activation, macrophages release nuclear factor (NF)-κB-dependent pro-inflammatory cytokines, such as interleukin (IL)-1 and tumor necrosis factor-α (TNF-α) (35). In sarcoidosis, BALF cells and monocytes highly express toll-like receptor (TLR)-2 (36, 37) and produce increased amounts of TNF-α, IL-1β, and IL-6 compared with controls, when stimulated with TLR-2 ligands, including ESAT-6 and KatG (7, 36–38). A role for TLR-2 in immune activation and granuloma formation in sarcoidosis is further supported by genetic and mouse studies (38, 39). Lately, continuous TLR-2 ligation by macrophage-derived serum amyloid A has been suggested to contribute to persistent stimulation of the immune response in sarcoidosis (37).

Intrinsically, unstimulated sarcoid-derived alveolar macrophages (AMs) produce increased amounts of IL-1 and TNF-α (40–43) and are highly activated (44, 45). The amounts of spontaneously produced TNF-α by BALF cells *in vitro* correlate with the presence of aggregates of AMs in the tissue (46, 47). Only AMs from patients with active and progressive disease produce increased amounts of TNF-α (48–51). These data highlight the role of TNF-α in granuloma formation and integrity, also supported by mouse studies (52–54).

Important mechanisms of action of TNF-α include macrophage activation, promotion of cellular migration toward the site of inflammation and leukocyte adhesion (52, 55, 56). In a mycobacterial-driven mouse model, TNF-α is responsible for the early production of chemokines that attract mononuclear cells to the site of inflammation, such as RANTES, MIP-1α, MIP-1β, MIP-2, and MCP-1 (55), of which increased amounts are found in sarcoidosis BALF (57–59). In active sarcoidosis, AMs produce high amounts of CCL20, when stimulated by TNF-α and IL-1β (60). CCL20 is a chemokine with high affinity for chemokine receptor CCR6, therefore attracting DCs, B cells, and specific T cell subsets toward the lungs (60, 61). Similarly, AM-derived CXCL10 and CXCL16 contribute to CXCR3^+^ and CXCR6^+^ CD4^+^ Th cell recruitment (62, 63).

In a mycobacterial-driven granuloma model, efficient cellular recruitment, mediated by AM-derived CXCL10 and CXCL16, depends on interferon-γ (INF-γ) (53). During the early innate response natural-killer (NK) cells are important producers of INF-γ, when stimulated by TNF-α, IL-1, and IL-12. In sarcoidosis, the size of a distinct NK cell subpopulation (CD56^bright^CD94^high^KIR^low^) is increased in the BALF compared with controls (64). Furthermore, higher proportions of NK cells were found to correlate with a poor outcome (65).

Once recruited, TNF-α is needed for leukocyte adhesion, since an abrogation of tightly formed granulomas in TNF-α-deficient mice is observed following mycobacterial infection (55). In sarcoidosis, TNF-α induced the expression of intracellular adhesion molecule-1 (ICAM-1) on AMs, leading to cellular aggregation (66). Additionally, leukocyte adhesion molecule (LeuCAM) expression, such as CD11a/b/c and CD18 (67), is increased in sarcoid AMs compared with controls.

Following adhesion, epithelioid histiocytes and monocyte-derived DCs (moDCs) can fuse to MGCs when stimulated by local cytokines, such as TNF-α, GM-CSF, IL-17A, CCL20, and INF-γ (68, 69). Patient-derived macrophages and monocytes show an enhanced potential to form MGCs *in vitro*, compared with healthy controls and other granulomatous diseases (70).

Importantly, sarcoid-derived AMs have an increased accessory function on autologous blood- and lung-derived T lymphocytes, when compared with controls (71–73). Macrophages are not capable to migrate to the LN to induce naive T cell activation, making them weak APCs. Nonetheless, in sarcoidosis, macrophages might contribute to local antigen presentation, enhancing proliferation of chemokine-recruited memory Th cells.

In summary, macrophages are important for the initial accumulation, aggregation, and fusion of the cellular building blocks needed for granuloma formation. This process is mediated by the strong immune modulatory capacities of TNF-α and assisted by NK cells, which produce INF-γ.

### Dendritic cells

Only a few studies investigated the role of DCs in sarcoid granuloma formation (47, 74). Our group has shown that granuloma formation surrounding intravenously injected antigen-loaded beads trapped in the lung vasculature is dependent on DC-initiated Th cell proliferation within the mediastinal LN (75). In sarcoidosis, an accumulation of mature (Fascin^+^HLA-DR^+^DC-LAMP^+^) DCs is found surrounding LN granulomas, adjacent to CD3^+^ lymphocytes, suggesting DC-T cell interaction at this site (76). Mature (CD11c^+^CD86^+^) DCs are found surrounding granulomas in sarcoid-derived mucosal biopsies (77), further supporting a role for DCs in airway and parenchymal granuloma formation.

An impaired accessory function of *ex vivo* blood-derived myeloid DCs (mDCs) has been suggested to contribute to granuloma formation, as clearance of the putative antigen may be ineffective and the immune system turns to granuloma formation as a default immunological response (78). In contrast, our group isolated BALF mDCs of sarcoidosis patients and found them to be immunocompetent, initiating proliferation of allogeneic, naïve T lymphocytes comparable with mDCs from healthy controls (77). Similarly, *in vitro* cultured moDCs showed a comparable accessory capacity as controls, although they are intrinsically prone to produce TNF-α (77, 79). Hence, it is most likely that DCs are involved in granuloma formation, instead of displaying diminished antigen-presenting capacities.

Differentiation of T lymphocytes depends on the local cytokines surrounding the initiating APC (80). Although it is very likely that LN-specific interactions, mediated by DCs, are responsible for the initial T cell polarization toward a Th1 and Th17 phenotype as observed in sarcoidosis, direct evidence is still lacking.

### Lymphocytes

Sarcoidosis is characterized as a Th1- (81) and more recently a Th17-mediated disease (61, 82), based on the accumulation of INF-γ, IL-2, and IL17-producing Th cells in the lungs of patients with active sarcoidosis (44, 61, 82–84).

Th1 differentiation depends on IL-12 and IL-18, which are increased in BALF of sarcoidosis patients (85, 86). Alveolar epithelial cells type II (AEC-II) may contribute to this Th1-favoring environment, since patient-derived AEC-II produce IL-18 upon TLR-2 stimulation (87, 88). Additionally, AEC-II may contribute to CXCR3^+^ Th1 cell recruitment by production of CXCL10 (89).

Th17 differentiation is driven by IL-6 and TGF-β, both produced by sarcoid-derived BALF cells (90, 91), whereas survival and proliferation of this subset is IL-23-dependent (92–94). Increased expression of the IL-23-receptor and IL-17, both expressed by Th17 cells, is found in blood-, lung-, and LN-derived lymphocytes of active sarcoidosis patients, and not in inactive disease (61, 82). Recently, ESAT-6-specific Th17 cells in the BALF of sarcoidosis patients were found (95). Additionally, IL-17A is essential for granuloma formation in the lung during mycobacterial infection (96) or in chronic granulomatous disease (97).

We recently found that the proportions of circulating IL-17A/IFN-γ and IL-17A/IL-4 double-producing cells are significantly increased in the peripheral blood of patients and are present in substantial numbers in BALF (82). Findings in several autoimmune diseases have indicated the pathogenic potential of CD4^+^ Th cells producing both IL-17 and IFN-γ (98, 99). Processes underlying Th17 cell induction in sarcoidosis remain obscure, but the presence of these cells can suggest a role for autoimmune responses in sarcoidosis. B lymphocytes and plasma cells are found surrounding sarcoid granulomas (100). Additionally, active sarcoidosis patients have increased serum levels of B-cell-activating factor (BAFF) (101). Since B cell maturation and function depends on BAFF, its aberrant expression can initiate defective selection of autoreactive B cells, leading to autoantibody production (101, 102). In sarcoidosis, approximately 30–60% of the patients exhibit antinuclear antibody (ANA) positivity (101, 103).

A SNP in the IL-23 receptor gene has been associated with chronic sarcoidosis (104), which may contribute to Th17 cell development in sarcoidosis. Since IL-23 is a heterodimer of the p19 subunit and the p40 subunit of IL-12 (105) the Th1- and Th17-promoting cytokines share a common therapeutical target. Ustekinumab, a neutralizing antibody against the IL-12/IL-23 p40, was shown to be successful in the Th1/Th17-mediated diseases psoriasis and Crohn’s disease (CD) (106, 107), but not in chronic pulmonary or skin sarcoidosis (108, 109).

## Granuloma Integrity

In the majority of the sarcoidosis patients, granulomas spontaneous resolve within several years, without need for therapy. However, a substantial proportion of the patients develop chronic progressive disease, whereby granulomas persist and form fibrotic lesions, leading to debilitating disease, and sometimes death (1, 110). The immunological response, determining granuloma sustainment is not well understood.

### Regulatory T cells

Regulatory T cells (Tregs) play an important role in diminishing Th cell specific responses and are pivotal for maintenance of self-tolerance and immune homeostasis (111). An impaired immunosuppressive function of sarcoid-derived Tregs has been suggested to contribute to the on-going, exaggerated immune response, since sarcoid blood-derived (CD4^+^CD25^high^) Tregs fail to inhibit granuloma growth in an *in vitro* granuloma culture model (112, 113). Subsequently, an impaired immunosuppressive function of both blood- and BALF-derived sarcoidosis Tregs has repetitively been described on autologous and allogeneic healthy Th cell proliferation (114–116). These studies also show that sarcoid-derived Tregs fail to inhibit production of TNF-α, INF-γ, and IL-2, contributing to granuloma formation, rather than diminishing the immune response (112, 113, 116). It remains unknown what mechanism(s) underlies this impaired function.

Active and persisting sarcoidosis was recently associated with a global CD4^+^ T cell subset dysfunction (116). Notably, both Th anergy and Treg malfunctioning were restored in patients with disease resolution (116). These results highlight the complex interplay between pro-inflammatory and anti-inflammatory responses needed for granuloma integrity. This fine balance may explain contradictory results with regard to reported Treg numbers in the BALF (112–119) (Table 1). Low BALF Tregs (i.e., less immunosuppression) in patients have been associated with a favorable prognosis in a Scandinavian population (118). In contrast, a German study reported decreased BALF Treg numbers in sarcoidosis patients who develop chronic (active) disease, when compared with controls and patients who develop spontaneous resolution (115). Similarly, CD1d-restricted natural-killer T (NKT) cells with immunoregulatory function are greatly reduced in the peripheral blood of all sarcoidosis patients, except Löfgren patients (120).

**Table 1:** **An overview of studies reporting regulatory T cell (Treg) proportions and functional properties in pulmonary sarcoidosis**.

Study	Methods			Proportions			Function			Remarks
	Population	Treg definition	Technique	Blood	BALF	LN	Blood	BALF	LN	
Miyara et al. (112)	Active disease	CD4^+^CD25^+^	FC/IHC	**↑**	**↑**^b^	**↑**	**↓**^∧^	=^b^	=	^∧^Blood-derived Tregs reduce autologous T cell proliferation similarly as controls, but do not inhibit the release of TNF-alpha and INF-y
		% of CD4^+^								
Idali et al. (117)	Active disease	CD4^+^FoxP3^+^	FC/PCR	**↓**	**↓**					BALF Treg proportions are significantly higher than blood Treg proportions in both healthy controls and patients
		% of CD4^+^								
Taflin et al. (113)	Active disease	CD4^+^CD45RA^−^FoxP3^++^	FC/IHC	**↑**		**↑**^b^	**↓**			FoxP3^+^ Tregs in the sarcoid LN are highly proliferative (Ki67^+^)
		% of CD4^+^								
Prasse et al. (115)	Pre-treatment patients	CD4^+^CD25^+^CD127^−^% of CD4^+^	FC		**↓**^∧^			**↓**,#^c,#^		^∧^BALF Treg proportions are decreased in patients who develop active chronic disease, defined after 1 year follow-up
										^#^Vasoactive intestinal peptide (VIP) inhalation increased the number of BALF Tregs and the immunosuppressive function
Rappl et al. (114)	Unknown	CD25^+^CD7^−^% of CD4^+^ CD45RO^+^ FoxP3^+^CD127^−^	FC		**↑**		**↓**			Increased proportions of CD4^+^ FoxP3^+^ CD127^−^Tregs are CD7-, compared with healthy controls
Wiken et al. (118)	Active disease^a^	FoxP3^+^	FC		**↓**^d^					BALF Tregs proportions are siginificantly decreased in HLA-DRB1*0301 positive patients, which are mostly (82%) Lofgren patients
		% of CD4^+^CD45RO^+^ CD27^+^								
Darlington et al. (119)	Active disease	CD4^+^FoxP3^+^	FC			=^b^				% FoxP3 expressing CD4^+^ T cells is inversely correlated with% T cells with AV2S3 >10% in BALF
		% of CD4^+^								
Oswald-Richter et al. (116)	Active disease	CD4^+^CD45RO^+^ CD25^high^	FC	**↑**			**↓**			Treg malfunctioning restored during disease resolution
		% of CD4^+^								

All results are compared with healthy controls, unless specified otherwise. Flowcytometry (FC), Immunohistochemistry (IHC), Polymerase chain reaction (PCR), broncho-alveolar lavage fluid (BALF), lymph node (LN).

^a^HLA-DRB1*0301 positive sarcoidosis patients were analyses vs. HLA-DRB1*0301 negative sarcoidosis patients.

^b^Compared with diseased controls.

^c^Compared with post-treatment.

^d^Compared with HLA-DRB1*0301 negative patients.

Taken together, these studies imply different roles for immune regulatory cells in sarcoidosis, either contributing to or preventing an on-going, exaggerated immune response. Arguably, whereas in the early sarcoid response there may be no need for Tregs to inhibit an effective immune response, during persistent stimulation immune regulatory cells should function as a natural brake on the exaggerated response to prevent immunopathology and autoimmunity.

### Interfering with granuloma integrity

Effective treatment agents used for sarcoidosis interfere with granuloma integrity and would ideally prevent fibrogenesis. Glucocorticosteroids (GCs), the main stay of sarcoidosis therapy, partially exert their beneficial effect by repression of NF-κB-related cytokine gene transcription and induction of lymphocyte apoptosis (121, 122). Using a mouse model, Tregs are found to be less sensitive to GC-induced apoptosis compared with Th cells, favoring an anti-inflammatory milieu (123, 124). Similarly, anti-TNF agents induce monocyte and lymphocyte apoptosis (125–127), while improving Treg numbers (123). Interestingly, infliximab, which blocks membrane-bound TNF-α, is uniquely associated with a high risk of reactivation of latent *Mtb* infection, whereas etanercept, solely blocking secreted TNF-α, is not (29). This phenomenon implies a critical role of membrane-bound TNF-α signaling in granuloma integrity (29), which is further supported by mouse studies (128).

Whether GCs and anti-TNF agents interfere with the delicate Th/Treg balance in pulmonary sarcoidosis, remains to be elucidated. Research into this field will shed more light on the role of Tregs in sarcoid pathology and whether Treg induction holds a promising new therapeutical strategy. Finally, an interplay between anergic Th cells, IL-10, alternatively activated macrophages (M2), CCL18 and lung fibroblasts has recently been suggested to contribute to fibrotic remodelling of the lung in chronic sarcoidosis (129). These insights yield new therapeutical targets to prevent irreversible organ damage in chronic pulmonary sarcoidosis patients.

## Conclusion

Sarcoidosis is an intriguingly complex granulomatous disorder, characterized by an exaggerated Th1/17 immune response, initiated by APCs, and maintained due to malfunctioning of Tregs. Refining insight into immunological events that determine granuloma fate may help identify new therapeutical targets and patients who will benefit such therapy in the future.

## Conflict of Interest Statement

The authors declare that the research was conducted in the absence of any commercial or financial relationships that could be construed as a potential conflict of interest.
